# Supplementary material for: Chemical evolution of primordial salts and organic sulfur molecules in the asteroid 162173 Ryugu
Source: Nat Commun. 2023 Sep 18;14:5284. doi: 10.1038/s41467-023-40871-0 (PMC10507048; doi:10.1038/s41467-023-40871-0)
Supplement: Supplementary file 1 — Supplementary Information [file 41467_2023_40871_MOESM1_ESM.pdf]

Supplementary Information for

"Chemical evolution of primordial salts and organic sulfur molecules in the asteroid 162173 Ryugu"

Toshihiro Yoshimura[1*], Yoshinori Takano[1*], Hiroshi Naraoka[2], Toshiki Koga[1], Daisuke Araoka[3], Nanako O. Ogawa[1], Philippe Schmitt-Kopplin[4,5], Norbert Hertkorn[4], Yasuhiro Oba[6], Jason P. Dworkin[7], José C. Aponte[7], Takaaki Yoshikawa[8], Satoru Tanaka[9], Naohiko Ohkouchi[1], Minako Hashiguchi[10], Hannah McLain[7], Eric Thomas Parker[7], Saburo Sakai[1], Mihoko Yamaguchi[11], Takahiro Suzuki[11], Tetsuya Yokoyama[12], Hisayoshi Yurimoto[13], Tomoki Nakamura[14], Takaaki Noguchi[15], Ryuji Okazaki[2], Hikaru Yabuta[16], Kanako Sakamoto[17], Toru Yada[17], Masahiro Nishimura[17], Aiko Nakato[17], Akiko Miyazaki[17], Kasumi Yogata[17], Masanao Abe[17], Tatsuaki Okada[17], Tomohiro Usui[17], Makoto Yoshikawa[17], Takanao Saiki[17], Satoshi Tanaka[17], Fuyuto Terui[18], Satoru Nakazawa[17], Sei-ichiro Watanabe[10], Yuichi Tsuda[17], Shogo Tachibana[17,19], Hayabusa2-initial-analysis SOM team[#]

---

[1] Biogeochemistry Research Center (BGC), Japan Agency for Marine-Earth Science and Technology (JAMSTEC), Natsushima 2-15, Yokosuka, Kanagawa 237-0061, Japan.

[2] Department of Earth and Planetary Sciences, Kyushu University, 744 Motooka, Nishi-ku, Fukuoka, 819-0395, Japan.

[3] Geological Survey of Japan (GSJ), National Institute of Advanced Industrial Science and Technology (AIST), 1-1-1 Higashi, Tsukuba, Ibaraki 305-8567, Japan.

[4] Helmholtz Zentrum München, Analytical BioGeoChemistry, Ingolstaedter Landstrasse 1, 85764 Neuherberg, Germany.

[5] Technische Universität München, Analytische Lebensmittel Chemie, Maximus-von-Forum 2, 85354 Freising, Germany.

[6] Institute of Low Temperature Science (ILTS), Hokkaido University, N19W8, Kita-ku, Sapporo, 060-0189, Japan.

[7] Solar System Exploration Division, NASA Goddard Space Flight Center, Greenbelt, Maryland 20771, U.S.A.

[8] HORIBA Advanced Techno, Co., Ltd., Kisshoin, Minami-ku Kyoto, 601-8510, Japan.

[9] HORIBA Techno Service Co., Ltd. Kisshoin, Minami-ku Kyoto, 601-8510, Japan.

[10] Department of Earth and Planetary Sciences, Nagoya University, Nagoya 464-8601, Japan.

[11] Thermo Fisher Scientific Inc., 3-9 Moriyacho, Kanagawa-ku, Yokohama-shi, Kanagawa 221-0022, Japan.

[12] Department of Earth and Planetary Sciences, Tokyo Institute of Technology, Ookayama, Meguro, Tokyo, 152-8551, Japan.

[13] Creative Research Institution (CRIS), Hokkaido University, Sapporo, Hokkaido, 001-0021, Japan

[14] Department of Earth Science, Tohoku University, Sendai, 980-8678, Japan.
[15] Department of Earth and Planetary Sciences, Kyoto University, Kyoto, 606-8502, Japan.
[16] Earth and Planetary Systems Science Program, Hiroshima University, Higashi Hiroshima, 739-8526, Japan.
[17] Institute of Space and Astro-nautical Science, Japan Aerospace Exploration Agency (ISAS/JAXA), Sagamihara, Kanagawa, 229-8510, Japan.
[18] Kanagawa Institute of Technology, Atsugi 243-0292, Japan.
[19] UTokyo Organization for Planetary and Space Science (UTOPS), University of Tokyo, Bunkyo-ku, Tokyo, 113-0033, Japan.
[#] A list of authors and their affiliations appears at the end of the paper

* equal contribution

Correspondence to T. Yoshimura: e-mail: yoshimurat@jamstec.go.jp, TEL: +81-46-867-9783

Contents

- List of Abbreviations
- Mineral dissolution in sequential leaching
- Comparison of cation and δ^{26}Mg compositions with those of carbonaceous meteorites
- Depth difference of NH_3 concentrations between Ryugu and Orgueil
- Supplementary Figures 1–10.
- Supplementary Table 1–8.
- Supplementary References 1–35.
- List of Hayabusa2-initial-analysis SOM team members

List of Abbreviations

C-type, Carbonaceous-type; CI, C-Ivuna type; CIA, Chemical Index of Alteration; CM, Mighei-type; CR, Renazzo-type; IC/OrbitrapMS, Ion chromatography/Orbitrap mass spectrometry; ESI, electrospray ionization; ICP-MS, Inductively Coupled Plasma mass spectrometry; SOM, Soluble Organic Matter; M–C–NK, Magnesium–Calcium–Sodium and Potassium; A–CN–K, Aluminum–Calcium + Sodium–Potassium.

Mineral dissolution in sequential leaching

In sequential leaching, mineral phases other than the main target minerals are also partly dissolved according to their solubility. For this study, we have verified extraction yields of acid dissolution by using standard materials of the main target minerals (Supplementary Table 5).

First, dolomite is the main carbonate mineral of Ryugu as well as other CI chondrites. We conducted an experiment in which dolomite standard published by the National Institute of Advanced Industrial Science and Technology (AIST) reacted with formic acid used in this study under the same conditions. The dissolution rate was calculated from the Ca concentration in the HCOOH after the reaction, and dolomite yield was 103.3 ± 5.0% (2SD, n = 3), indicating that it was completely decomposed.

The solubility of clay minerals in HCOOH and HCl was verified using reference materials of the Clay Science Society of Japan (CSSJ). According to our experiment, almost all of the saponite dissolved in 20% HCl (please see attached table below). Other phyllosilicates, montmorillonite, dickite, kaolinite, and pyrophyllite were also tested in the same way. Dissolution of clay minerals was evaluated by aluminum or magnesium concentration. The dissolution rate of saponite was 103.3 ± 2.2% (2SD, n = 3). Although the concentrations varied slightly around 100%, the JCSS elemental reference values themselves differ by 8.0% (2SD, n = 3) among the three different laboratories[1], so basically a 100% yield was achieved. The >99% HCOOH, which is the extraction fraction before #10 HCl, also dissolves about 10% of the saponite. The SOM extraction method of Naraoka et al. (2023)[2] used very concentrated formic acid, but this dissolution behavior of this clay mineral corresponds to the acidity of the monocarboxylic acid according to the test using 6.0% (1M) CH_3COOH (Supplementary Table 5).

The Fe concentrations of 0.16 and 0.37 mmol/g in A0106 and C0107 (Supplementary Table 2). If we assume that the amount of S measured in the #7-1 is all come from FeS, the amount of S released is about 1/600 and 1/400 of the total amount of SO_4^{2-} and $S_2O_3^{2-}$ in the #7-1 fractions of A0106 and C0107, respectively. Thus, the effect of sulfide dissolution on the composition of dissolved species in the solution is small. Sulfide dissolution mainly occurred in the fractions #10 HCl as well as in #9 HCOOH. Approximately ~1000 to 8000 times more

Fe is detected in the HCOOH and HCl fractions, respectively, compared to #7-1 hot H_2O fraction. In addition, the calculated Mg# (Mg/Mg+Fe) for #7-1 fractions of Ryugu is ~82, which is close to the typical value for Ryugu phyllosilicates (ranging mainly from 75 to 90)[3]. Iron may be derived from secondary minerals other than FeS, in which case the effect of sulfide dissolution on sulfur would be smaller.

Comparison of cation and δ^{26}Mg compositions with those of carbonaceous meteorites

To check the ionic balance of each fraction, the total ionic contents of major anions (Σ^-) and major cations (Σ^+) are expressed in meq (milliequivalent, the sum of moles of cation/anion multiplied by its valence): the sum of cations should equal the sum of anions (Supplementary Table 4). Because of their high solubility, Na and K ions should preferentially remain behind in concentrated brines, resulting in the Na/Mg and K/Mg ratios of the salt fraction being two to three orders of magnitude higher than those of the carbonate and phyllosilicate fractions (Supplementary Fig. 3). Alkali metal ions are preferentially partitioned over Mg, one of the most abundant soluble ions in meteorites, during their precipitation into secondary phases. The total salt concentration of Orgueil is higher than that of Ryugu, particularly that of Mg, which is more than 22 times higher in Orgueil than in the Ryugu samples.

For the carbonate and phyllosilicate fractions, some representative terrestrial materials are plotted on the ternary diagram of Al_2O_3, CaO+Na_2O, and K_2O, known as the A–CN–K compositional space[4], to describe the modes of mineral alteration (Supplementary Fig. 9). A considerable variation in solute compositions is present in the HCOOH carbonate extracts of Orgueil, Tarda, Aguas Zarcas, and Jbilet Winselwan, suggesting variable degrees of precipitation of carbonate minerals and ion-exchange processes. The Ryugu carbonate fractions show an intermediate composition.

The Ryugu phyllosilicate fractions are characterized by an ionic strength 2.5 to 5 times greater than those of the salt and carbonate fractions, reflecting a higher abundance of Mg- and Fe-enriched phyllosilicates and clays (Supplementary Table 4). Their A–CN–K compositions (Supplementary Fig. 9) are close to the cosmic abundance and the compositions of bulk CI, CV, and CM chondrites[5-7]. The CI1 Orgueil, CM2 Aguas Zarcas, and CM2 Jbilet Winselwan follow a path toward higher Al_2O_3 on the ternary diagram (Supplementary Fig. 9), indicating a greater amount of soluble element-depleted clays, e.g., smectite. The Mg, Ca, and Na+K compositions, hereafter the M–C–NK space, of Ryugu also lie close to those of the cosmic abundance[8] and CI, CV, and CM chondrites, as well as ordinary chondrites[5-7] (Fig. 2). Both the A–CN–K and M–C–NK compositions of the Ryugu phyllosilicate fractions can be regarded as relatively unaltered.

Both the bulk or leaching fractions from all meteorites show a linear trend along the A–CN junction of the A–CN–K triangle (Supplementary Fig. 9), indicating that there was no

transfer of incompatible elements to outside the meteorites, and that aqueous alteration occurred in a closed system. Here, the chemical index of alteration (CIA) is defined as:

$$CIA = [Al_2O_3/(Al_2O_3 + CaO^* + Na_2O + K_2O)] \times 100$$

where CaO* represents Ca in silicate-bearing minerals only[9]. The CIA is a quantitative indicator of the *in situ* production of clay minerals and is equivalent to projecting the plots of the ternary diagram onto the vertical scale to the left of the diagram (Supplementary Fig. 9). The extent of alteration in CM2 has previously been quantified by using the Fe^{3+}/Si ratio in the matrix phyllosilicate: this quantification is called the Mineralogical Alteration Index[10]. For the phyllosilicate fractions of meteorites, which were decarbonated by HCOOH, high CIA values mainly reflect removal of mobile Na and Ca relative to immobile Al during aqueous alteration of HCl-soluble clay fractions. CM2 chondrites have fully hydrated matrices, and the phyllosilicate fractions of Aguas Zarcas and Jbilet Winselwan have the highest CIA values of ~70, followed by Orgueil (~55). It should also be noted that the extent of partitioning in soluble cations of phyllosilicate fractions is subtle in the M–C–NK space; thus, element partitioning mainly occurs between immobile and mobile elements.

The three different solvent extracts display a large Mg isotopic (δ^{26}Mg) variation of 1.4‰ (Supplementary Fig. 6, Supplementary Table 8). Because the weighted δ^{26}Mg average of soluble extracts was nearly equal to that of the bulk chondrite composition, the difference in δ^{26}Mg reflects isotope fractionation during Mg partitioning by precipitation of salts, carbonates, and phyllosilicates. This study thus provides the first evidence that changes in aqueous alteration led to Mg isotopic heterogeneity for carbonaceous chondrites. The δ^{26}Mg values for the carbonate fractions are systematically lower than those of the phyllosilicate fractions by 0.3‰, except for Orgueil. The molar difference between the Mg contents of the Ryugu and Orgueil salt and carbonate fractions is probably attributable to Mg redistribution between those fractions, i.e., soluble salts and carbonates. Significant enrichment of sulfate in the Orgueil salt fraction cannot be explained by redistribution of sulfate from the carbonate and phyllosilicate fractions (Supplementary Table 2); thus, it is considered to derive from oxidation of FeNi sulfides. The M–C–NK composition of Orgueil also appears to have experienced a higher degree of alteration than the least-altered Na-rich Ryugu compositions. The stable magnesium isotope ratio (δ^{26}Mg) is a tool to differentiate primary aqueous alteration precipitates and later alteration processes. For the meteorites, except Orgueil, a 0.4‰ difference between the carbonate and phyllosilicate fractions mainly reflects isotope fractionation associated with carbonate precipitation. Given the opposite direction of isotopic fractionation between epsomite[11] and carbonates[e.g. 12] (dolomite; magnesite, $MgCO_3$), the differences in the Mg amounts and isotopic compositions of Orgueil compared to Ryugu are attributable to element redistribution between the salt and carbonate fractions. The phyllosilicate fraction of CI1

Orgueil has the highest δ^{26}Mg value of −0.07‰ ± 0.07‰[13,14], suggesting the more significant formation of ^{26}Mg-enriched clay compared to other petrologic type 2 chondrites (Supplementary Fig. 6).

Depth difference of NH_3 concentrations between Ryugu and Orgueil

Ryugu is reminiscent of Orgueil, but in addition to the differences in the salts, possesses different amounts and ratios of NH_3 and amines. One potential explanation for these differences is that Orgueil was more protected from volatile losses than Ryugu surface and subsurface materials, the latter of which were partly derived from ~1 m depth[15]. Preservation of labile organic compounds at the surface is affected by the balance between the rate of erosion, which determines the age of surface exposure, and solar/cosmic ray irradiation penetrating to depths of a few centimeters to a maximum of several meters[16]. If the prime difference between Ryugu and Orgueil were sampling depth, one would expect the properties of material from the subsurface collection in Chamber C to be bracketed by those of the surface collection in Chamber A and Orgueil (or Ivuna), but that does not seem to be the case.

Naraoka et al. (2023)[2] reported the presence of non-protein type amino acids and a wide variety of alkylated N-containing heterocyclic compounds (e.g., pyridine, pyrimidine, imidazole, and pyrrole) from the same sample (A0106). Parker et al. (2023) [17] also provided additional qualitative and quantitative detailed evaluation of indigenous amino acids as part of a comparison of the A0106 and C0107 samples. The diversity of N-heterocyclic molecules in carbonaceous meteorites is an important indicator of the origin and formation history of the parent body[18,19]. Initial descriptions of volatile nitrogen and noble gases in Ryugu samples has been reported from a detailed gas analysis[20,21].

In addition to organic-form nitrogen, ammonium salts and NH_4^+ adsorption/ion exchange with phyllosilicate minerals and complexation with organic matter are potential N reservoirs of meteorites[22]. The hydrophilic nature of N-containing functional groups, including amines, amides, and the imine moiety, makes them susceptible to aqueous alteration[23]; thus, N depletion of meteorites and comets is probably due to highly volatile N_2, NH_3, or possibly HCN[24]. In this context, it is important to note that the volatile organic nitrogen species methylamine (CH_3NH_2), ethylamine ($C_2H_5NH_2$), and isopropylamine and *n*-propylamine (both $C_3H_7NH_2$), together with small organic acids (i.e., HCOOH or CH_3COOH) have been detected in the A0106 and C0107 H_2O extracts[2,17]. The latter sample likely contains subsurface material from ejecta[15].

Compared to Orgueil, there is less extractable NH_3 in Ryugu (0.18 μmol/g for A0106 salt fraction and <0.02 μmol/g for C0107): Orgueil contains ~200 times as much NH_3 (33.54 μmol/g, see Supplementary Table 2). Either different amounts of terrestrial or aqueous alteration on Orgueil liberated bound NH_3 and amines[25], or the fluids in Ryugu and Orgueil had

different *p*H values. The depletion of NH_3 in the Ryugu (sub-)surface samples is also related to different chemical conditions of aqueous alteration. Higher-*p*H conditions resulted in the preferential formation of Cn–NH_2 ($n < 3$ for Ryugu A0106 and $n < 4$ for Orgueil) over NH_3 because its pK_a (25 °C) value of 10.6 is greater than that of NH_3 (9.3). At ~37 °C, the estimated temperature of the equilibrium point of Ryugu carbonate and magnetite[26], the pK_a of NH_3 in synthetic brines is 9.0 (ref. 27). Note that the pK_a of these species varies with temperature and ionic strength, so the formation of NH_3 salts might depend on (in decreasing order of importance), the *p*H, temperature, and ionic strength of the parent body fluids.

(A)

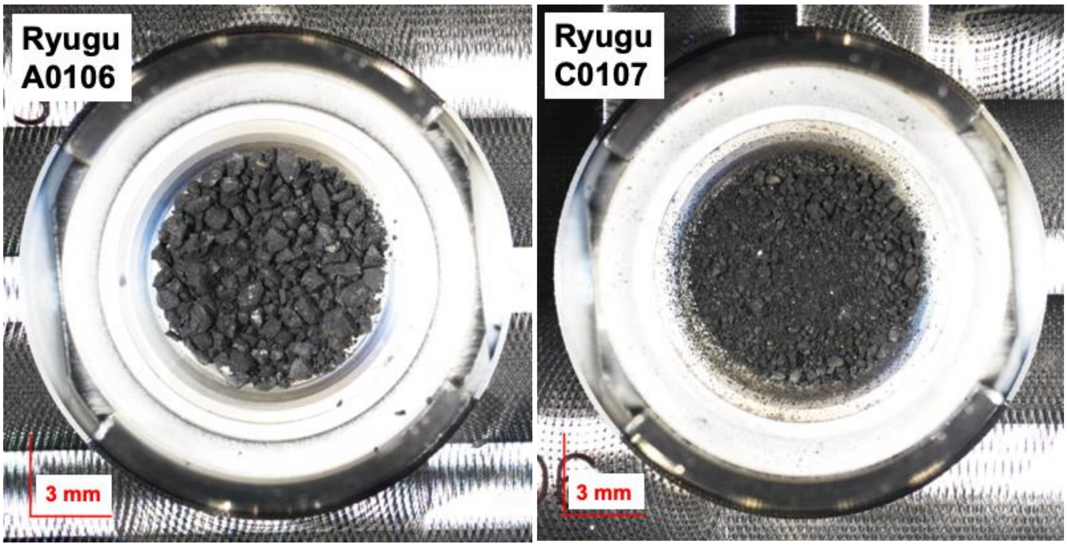

(B)

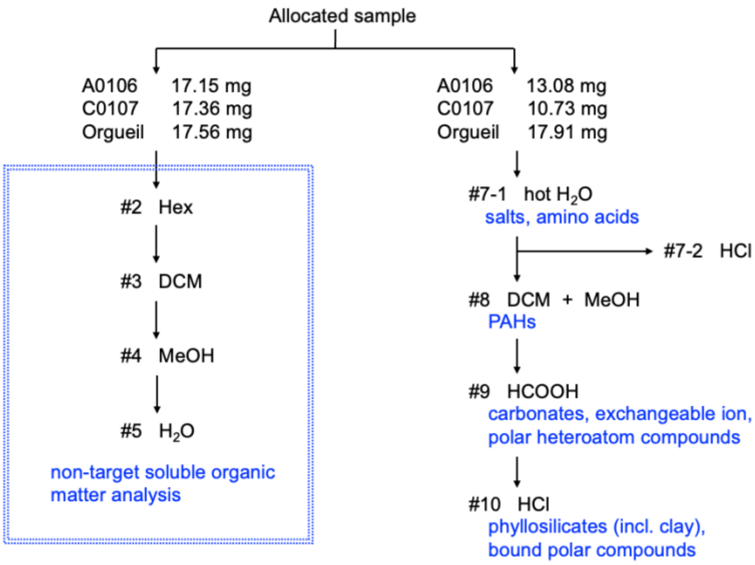

Supplementary Figure 1 Photograph and analytical scheme for the Soluble Organic Matter (SOM) of Ryugu samples.

(A) Photographs of the initial samples A0106 and C0107 from the asteroid Ryugu (162173), which were collected during the first and second touchdown samplings, respectively[15,28]. The photos were taken in the clean chamber of the JAXA curation facility prior to sample distribution. Details of the sample quality control and environmental assessments have been previously published[29,30,31]. Each red line of the scale bar represents 3 mm. **(B)** Analytical scheme for the initial analysis of Ryugu samples by the SOM team[2]

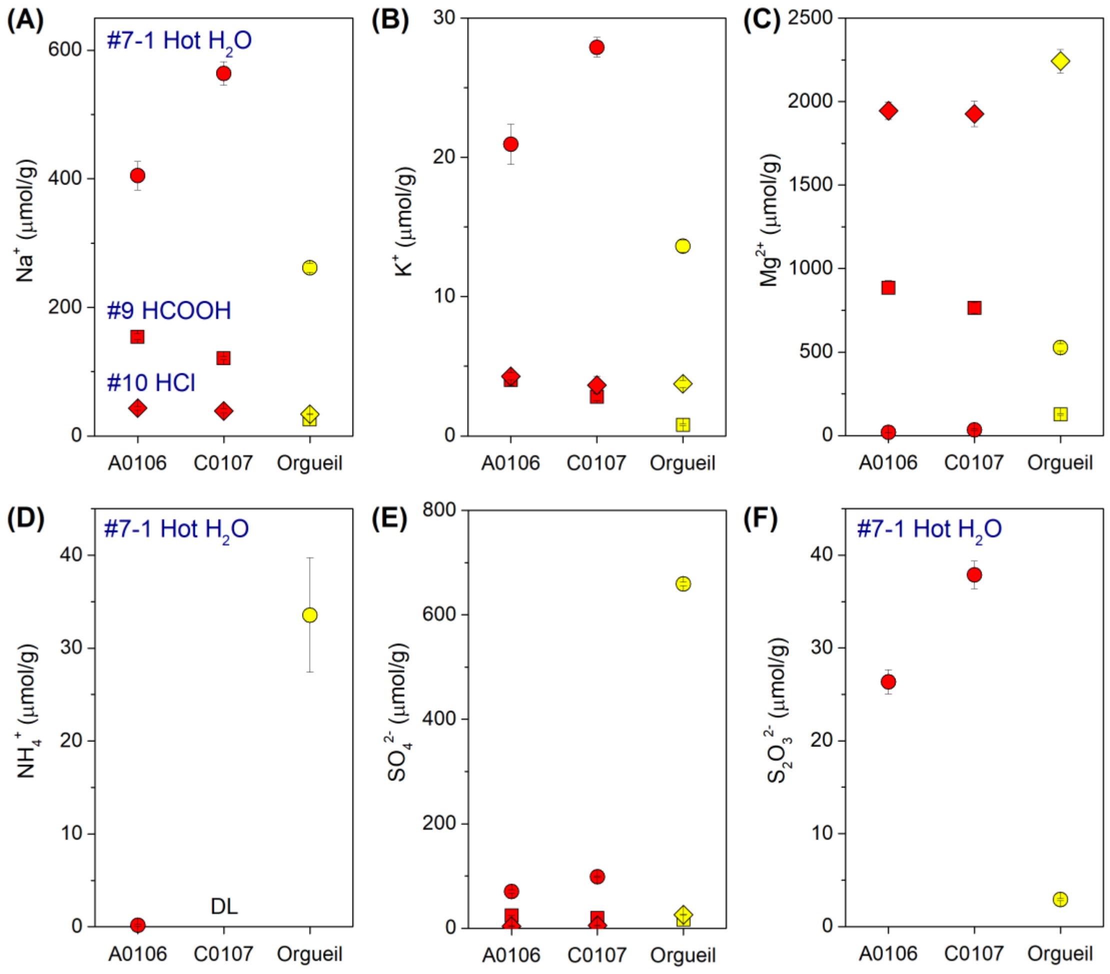

Supplementary Figure 2 The molar amounts of (A-D) cations and (E, F) anions in solute extracts divided by the initial weights of the starting solid materials used for sequential leaching (in µmol/g).

(A-C, E) Amounts of cations, Na^+, K^+, Mg^{2+}, and SO_4^{2-} in the salt fraction (via hot H_2O, circles), carbonate fraction (via HCOOH, squares), and phyllosilicate fraction (via HCl, diamonds), and **(D, F)** amounts of NH_4^+ and $S_2O_3^{2-}$ in the salt fraction. The extract numbers correspond to the original solvent identifications of Naraoka et al. (2023)[2]. DL is below the detection limit of the instrument, which is 0.02 µmol NH_4^+/g for hot H_2O fraction.

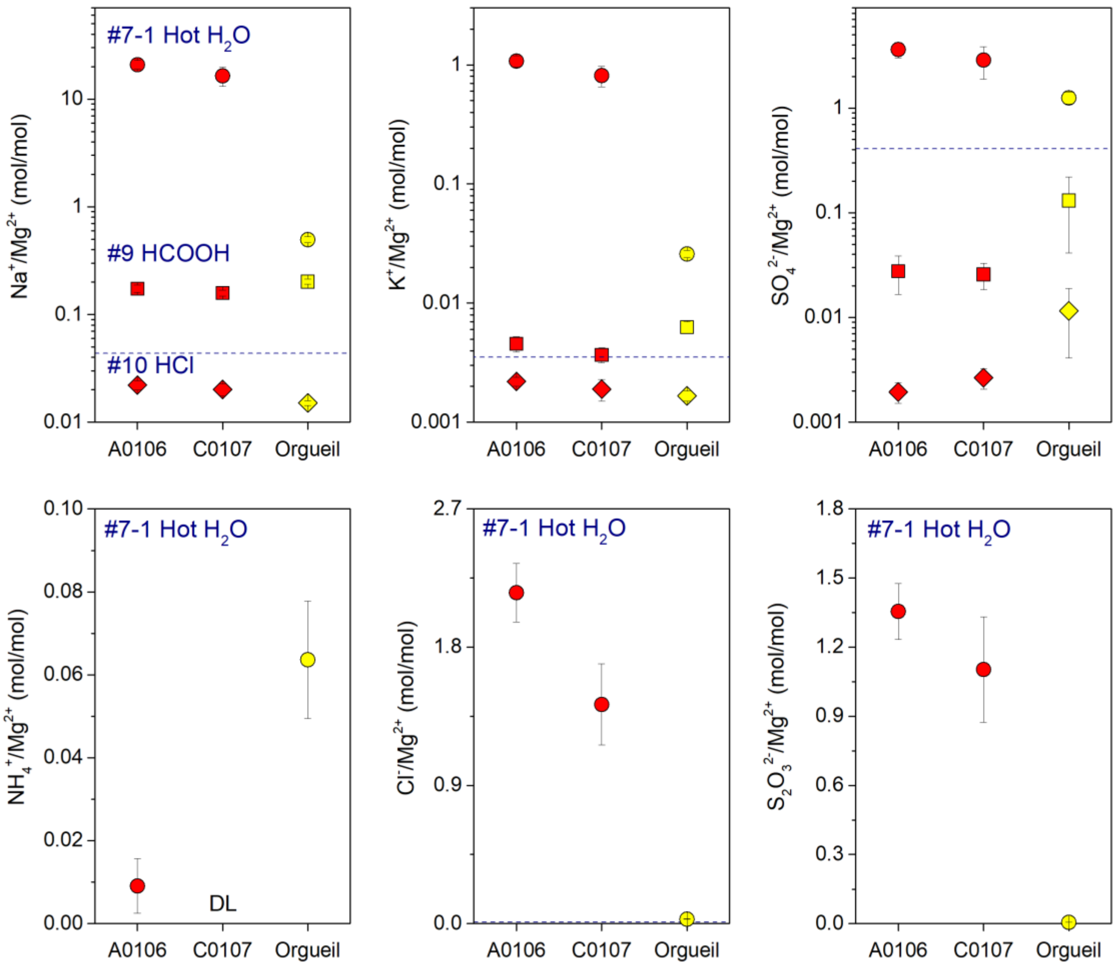

Supplementary Figure 3 Molar ratios of Na^+, K^+, SO_4^{2-}, NH_4^+, Cl^-, and $S_2O_3^{2-}$ to Mg^{2+} in the sequential extracts.

Na^+, K^+, and SO_4^{2-} to Mg^{2+} of the salt fraction (via hot H_2O, circles), carbonate fraction (via HCOOH, squares), and phyllosilicate fraction (via HCl, diamonds), plotted on a logarithmic scale, and the molar ratios of NH_4^+, Cl^-, and $S_2O_3^{2-}$ to Mg^{2+} in the salt fraction, plotted on a linear scale. Horizontal dashed lines represent the cosmic abundance values. The extract numbers correspond to the original solvent identifications of Naraoka et al. (2023)[2]. DL is below the detection limit of the instrument, which is 0.02 μmol NH_4^+/g for hot H_2O fraction. Data are provided as a Source Data file.

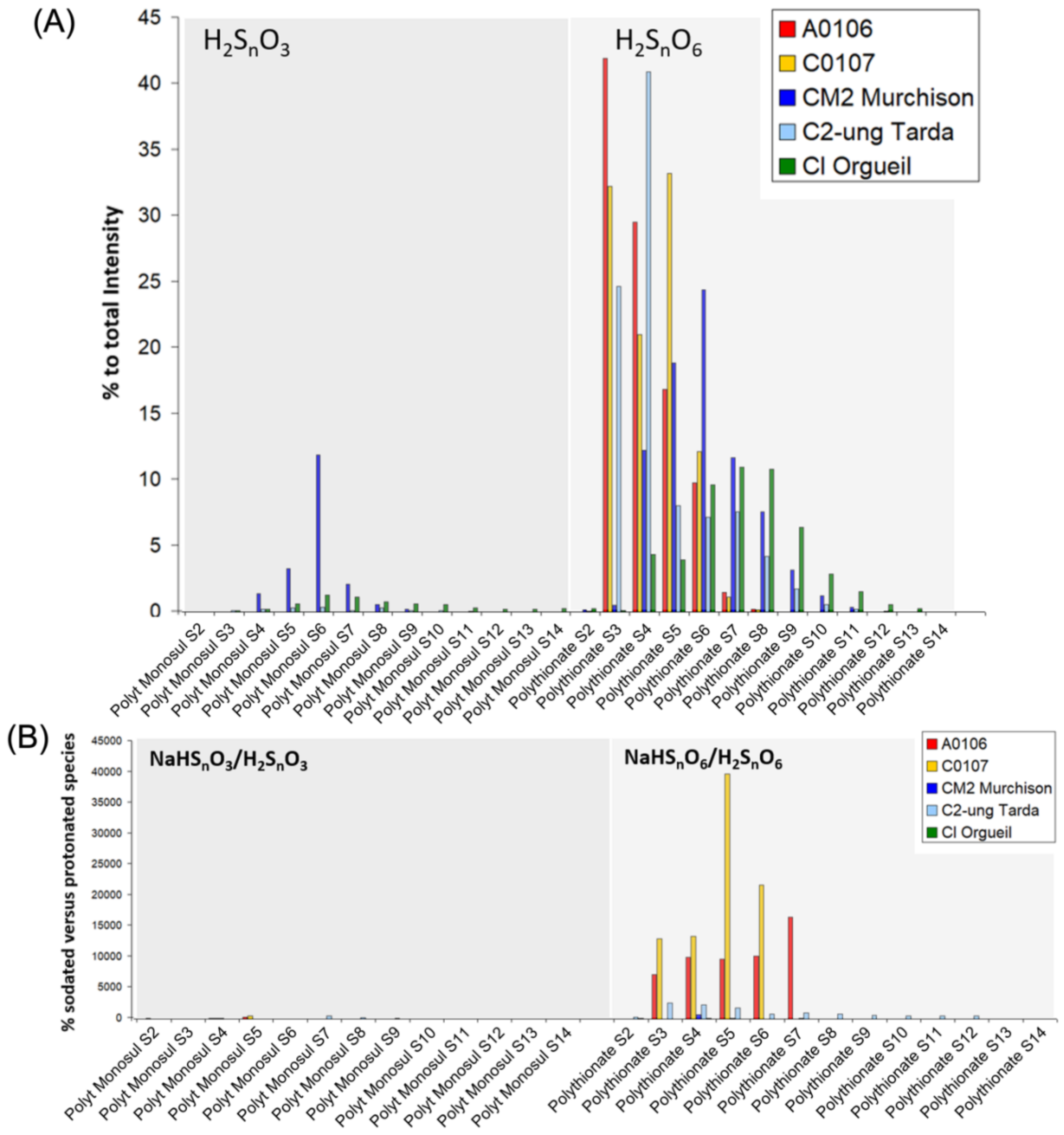

Supplementary Figure 4 Results of comprehensive analysis of sulfur-containing molecules using Fourier transform ion cyclotron resonance/mass spectrometry (FTICRMS).
(A) Percentage abundance of signal of A0106 and C0107 compared to Murchison, Tarda, and Orgueil meteorite methanol extracts. The relative intensities are the cumulative intensities of both the protonated and sodiated ions formed in the electrospray. **(B)** Abundance ratios of Na-adducts relative to the corresponding protonated species of the various polysulfides of A0106 and C0107 compared to Murchison, Tarda, and Orgueil meteorite methanol extracts. The analytical method followed Schmitt-Kopplin et al. (2010)[32].

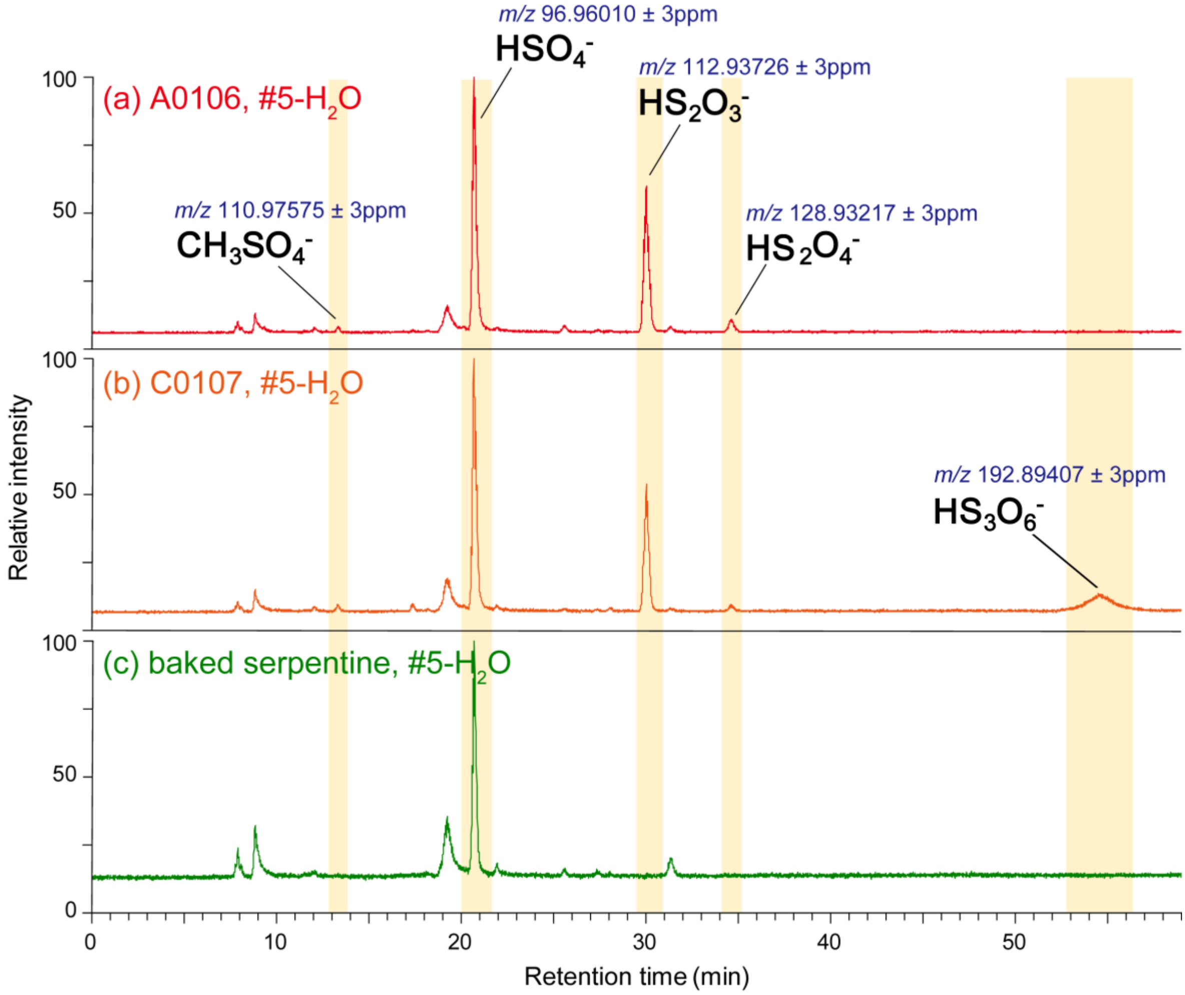

Supplementary Figure 5 Anion chromatogram of (a) the #5 water extracts of A0106, (b) C0107, and (c) baked serpentine obtained by ion chromatography/Orbitrap mass spectrometry in full scan mode.

The scans covered mass ranges of *m/z* 40–750. Major components are shaded.

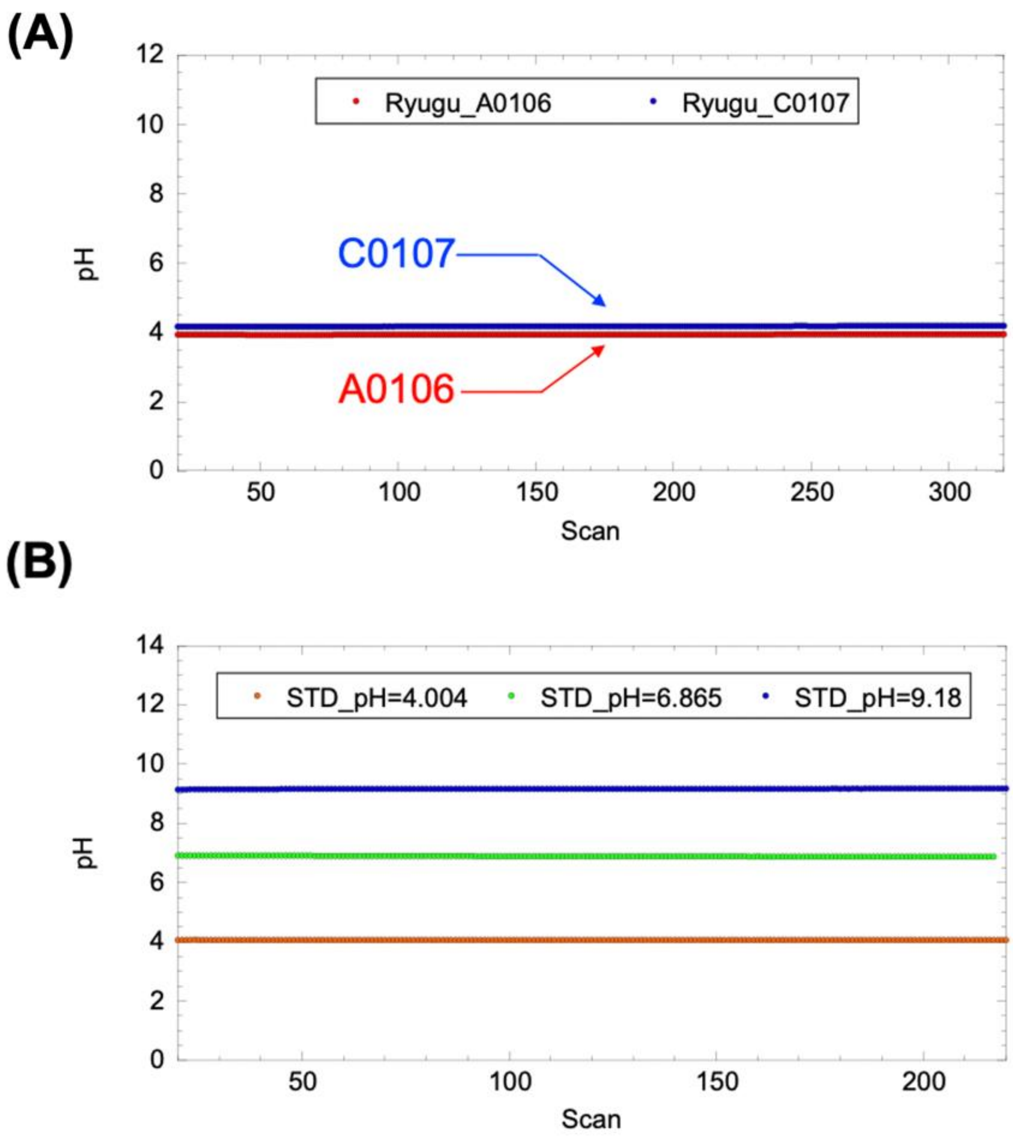

Supplementary Figure 6 The *p*H profiles for (A) hot H_2O extracts of Ryugu A0106 and C0107 and (B) working standard solutions (*p*H 4.004, 6.865, and 9.18).
The scan (*x*-axis) is each raw data integration, where the plateau represents the stabilized *p*H measurement status at a scan rate of 1 Hz.

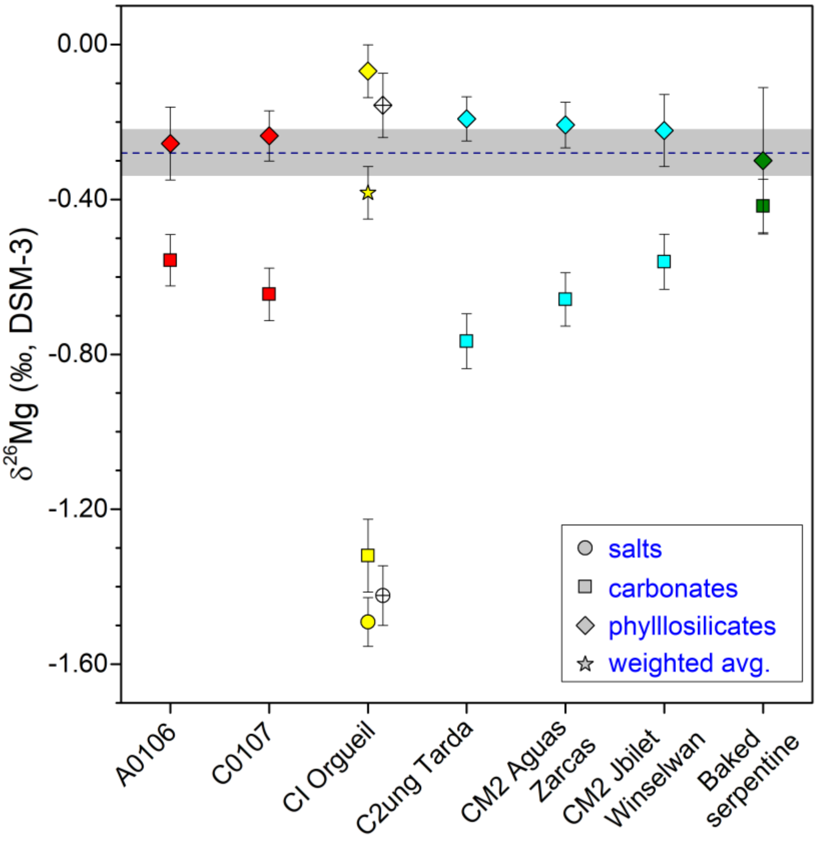

Supplementary Figure 7 Mg isotopic compositions of the sequential extracts.

The gray shading represents a δ^{26}Mg value of −0.28‰ ± 0.06‰ (vs. the DSM-3 standard[13]), which is the average value of nine types (CI, CM, CO, CV, L, LL, H, EH, and EL) of bulk chondrites. To confirm that an isotopic difference was present only in the Orgueil extracts, symbols containing a cross (#5 water and #7-2 HCl extracts) were extracted following different extraction orders than the other H_2O and HCl extracts (see Supplementary Fig. 1B). Both values are identical to those of #7-1 hot water and #10 HCl extracts (salt and phyllosilicate fractions, respectively) within analytical error. The HCl extract from CI1 Orgueil has the highest δ^{26}Mg value of −0.07‰ ± 0.07‰, suggesting more significant formation of ^{26}Mg-enriched clay compared to other petrologic type 2 chondrites. The weighted δ^{26}Mg average of the Orgueil soluble extracts was nearly equal to the bulk chondrite composition; therefore, their element redistribution associated with aqueous alteration occurred within a closed system.

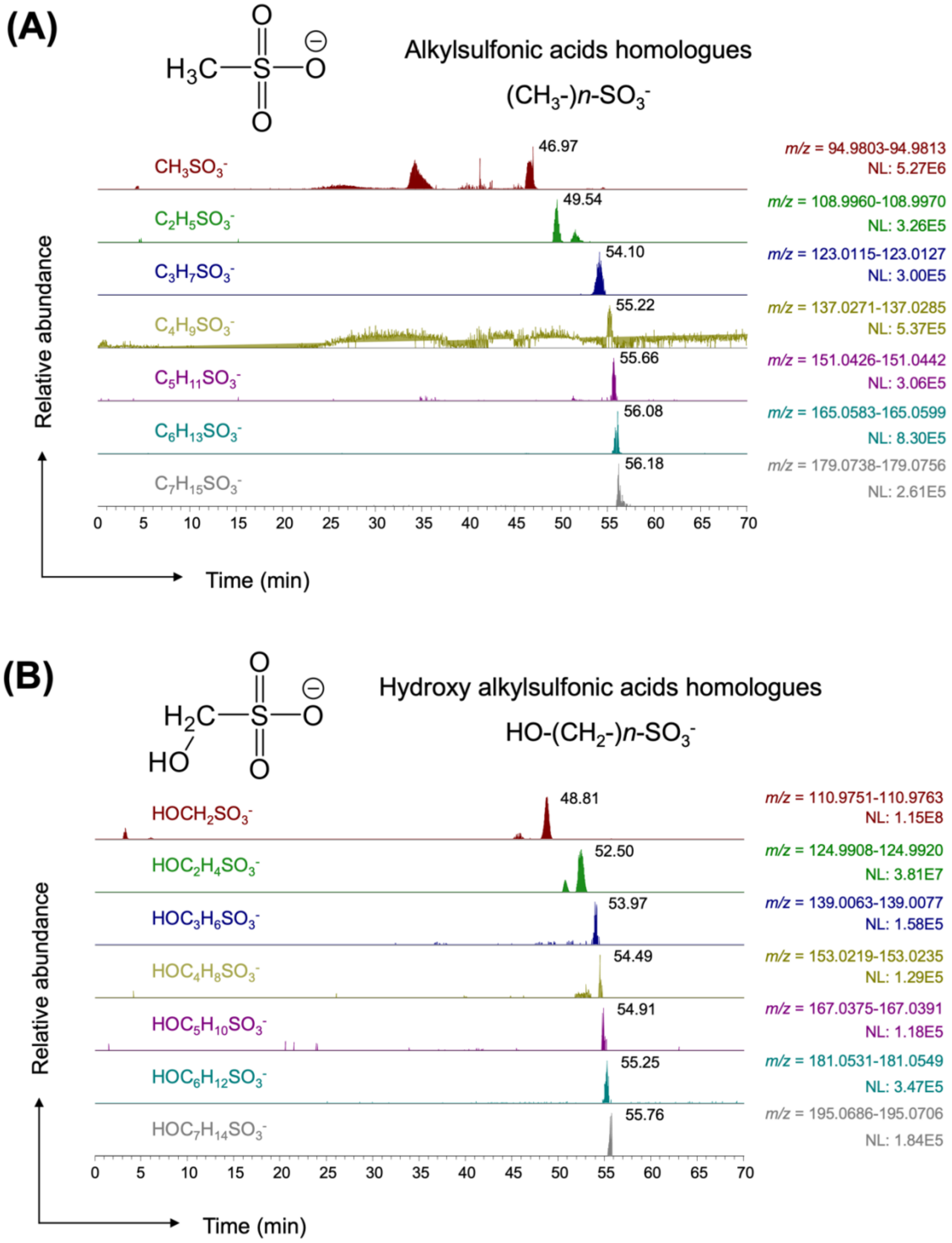
(A)
H3C—S—O
Alkylsulfonic acids homologues
(CH3-)n-SO3-
CH3SO3-
C2H5SO3-
C3H7SO3-
C4H9SO3-
C5H11SO3-
C6H13SO3-
C7H15SO3-
46.97
49.54
54.10
55.22
55.66
56.08
56.18
m/z = 94.9803-94.9813
NL: 5.27E6
m/z = 108.9960-108.9970
NL: 3.26E5
m/z = 123.0115-123.0127
NL: 3.00E5
m/z = 137.0271-137.0285
NL: 5.37E5
m/z = 151.0426-151.0442
NL: 3.06E5
m/z = 165.0583-165.0599
NL: 8.30E5
m/z = 179.0738-179.0756
NL: 2.61E5
Relative abundance
Time (min)
0 5 10 15 20 25 30 35 40 45 50 55 60 65 70
(B)
H2C—S—O
HO
Hydroxy alkylsulfonic acids homologues
HO-(CH2-)n-SO3-
HOCH2SO3-
HOC2H4SO3-
HOC3H6SO3-
HOC4H8SO3-
HOC5H10SO3-
HOC6H12SO3-
HOC7H14SO3-
48.81
52.50
53.97
54.49
54.91
55.25
55.76
m/z = 110.9751-110.9763
NL: 1.15E8
m/z = 124.9908-124.9920
NL: 3.81E7
m/z = 139.0063-139.0077
NL: 1.58E5
m/z = 153.0219-153.0235
NL: 1.29E5
m/z = 167.0375-167.0391
NL: 1.18E5
m/z = 181.0531-181.0549
NL: 3.47E5
m/z = 195.0686-195.0706
NL: 1.84E5
Relative abundance
Time (min)
0 5 10 15 20 25 30 35 40 45 50 55 60 65 70

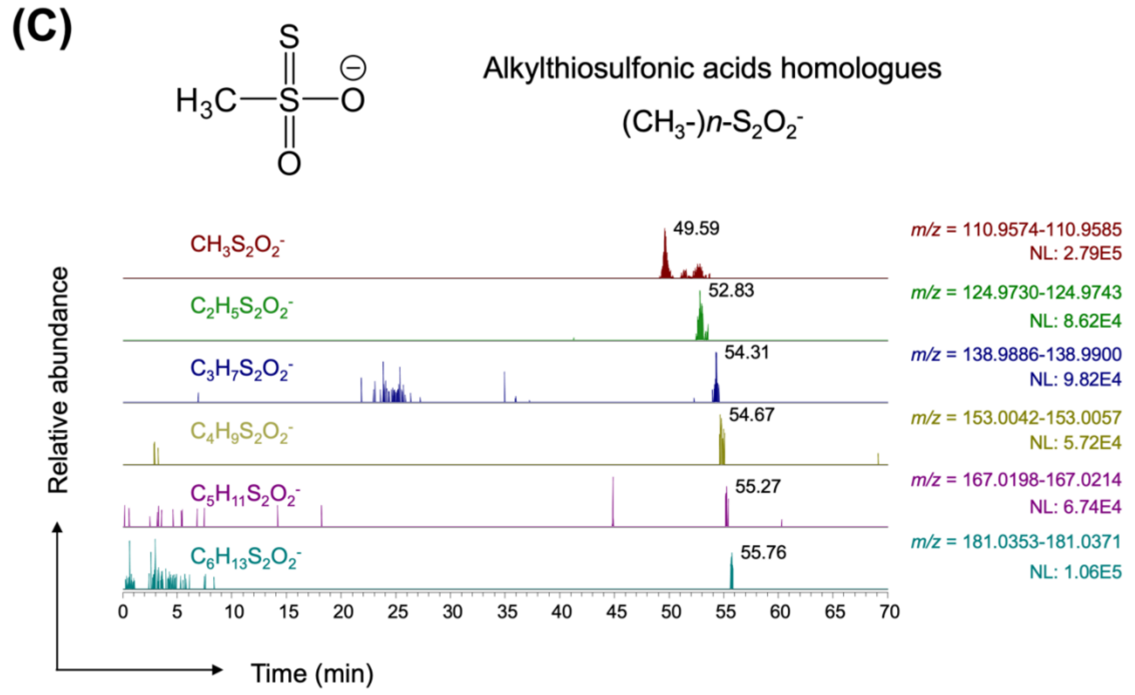

Supplementary Figure 8 Representative nanoLC/Orbitrap MS chromatograms of water-extractable (#5 H_2O extract) organic sulfur homologues obtained from the Ryugu A0106 sample.

(A) Alkylsulfonic acids; $(CH_3–)n–SO_3^–$, **(B)** hydroxyalkylsulfonic acids; $HO–(CH_2–)n–SO_3^–$, **(C)** alkylthiosulfonic acids; $(CH_3–)n–S_2O_2^–$.

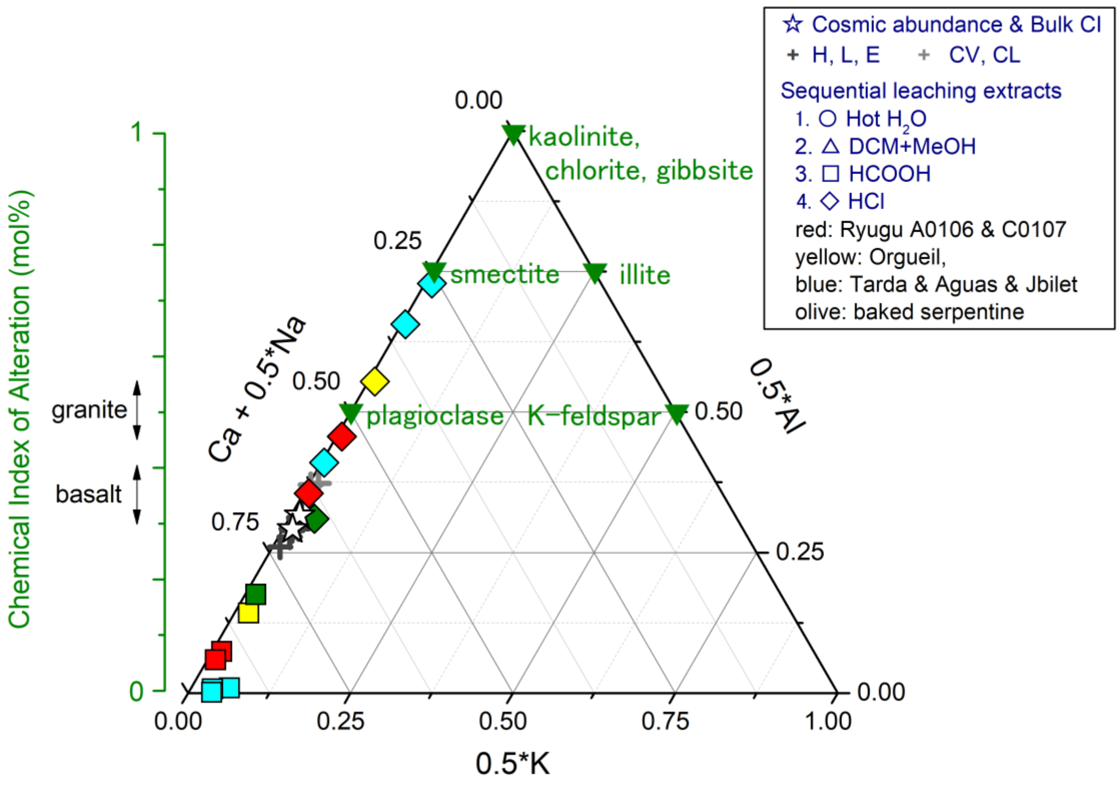

Supplementary Figure 9 Ternary diagram showing the molar proportions of Al_2O_3, CaO+Na_2O, and K_2O of the sequential extracts.

For reference, the bulk compositions of ordinary and carbonaceous chondrites, the cosmic abundance, and typical compositions of terrestrial rocks and minerals are also plotted. The left vertical scale is the chemical index of alteration, which is equivalent to projecting the plot of the A–CN–K ternary diagram onto the vertical scale to the left of the diagram. Data are provided as a Source Data file.

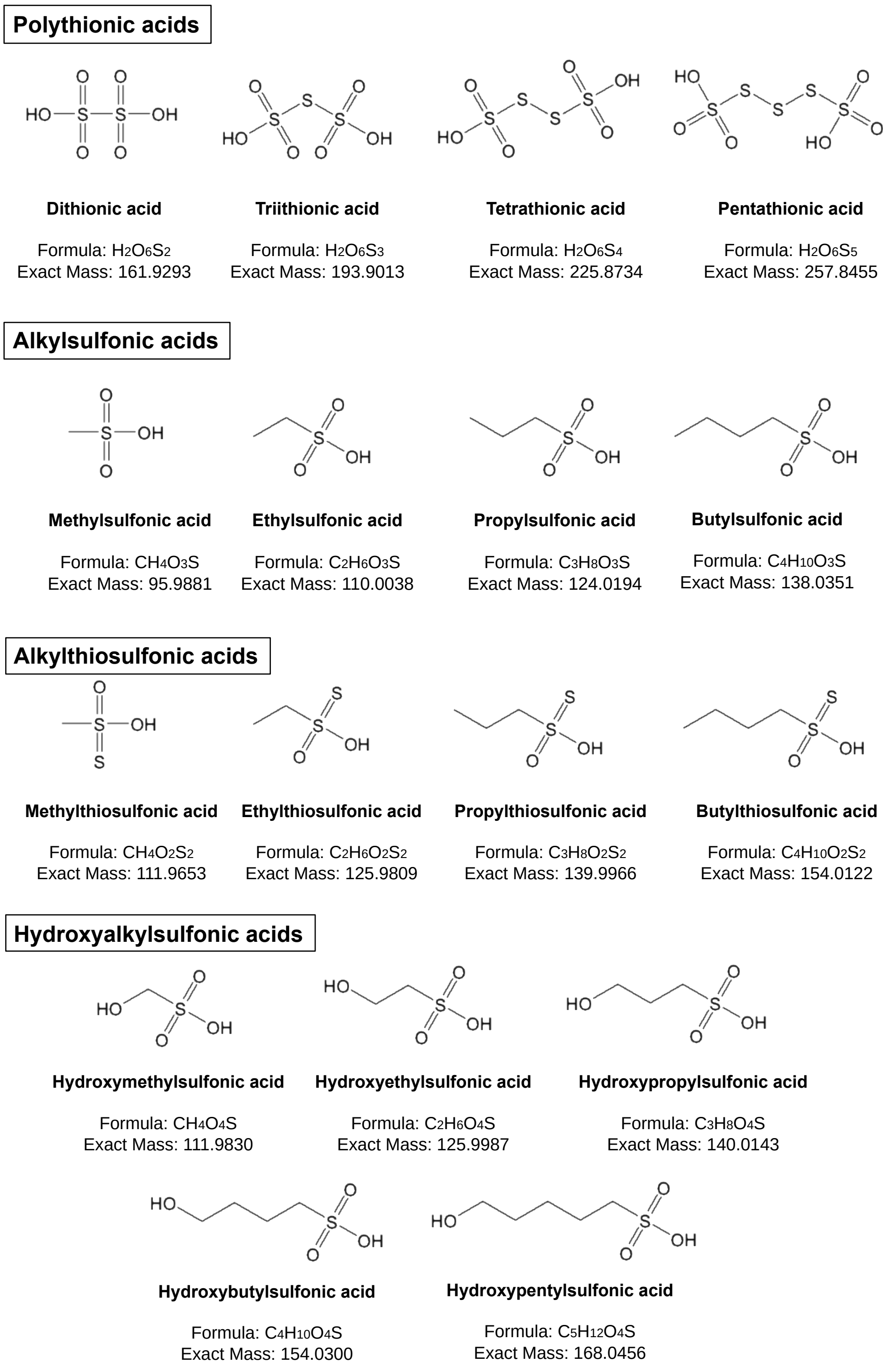

Supplementary Figure 10 Representative molecular structures of the polythionic acids and organosulfur compounds

(i.e., alkylsulfonic acids, alkylthiosulfonic acids, hydroxyalkylsulfonic acids, and hydroxyalkylthiosulfonic acids) discussed in this paper.

Hydroxyalkylthiosulfonic acids

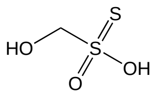

Hydroxymethylthiosulfonic acid

Formula: $CH_4O_3S_2$
Exact Mass: 127.9602

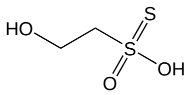

Hydroxyethylthiosulfonic acid

Formula: $C_2H_6O_3S_2$
Exact Mass: 141.9758

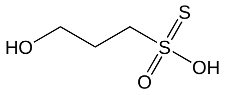

Hydroxypropylthiosulfonic acid

Formula: $C_3H_8O_3S_2$
Exact Mass: 155.9915

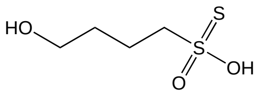

Hydroxybutylthiosulfonic acid

Formula: $C_4H_{10}O_3S_2$
Exact Mass: 170.0071

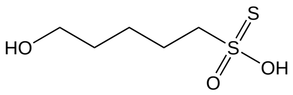

Hydroxypentylthiosulfonic acid

Formula: $C_5H_{12}O_3S_2$
Exact Mass: 184.0228

Supplementary Figure 10 (cont.)

Supplementary Table 1 Weights of the subsamples of A0106 and C0107 submitted for analysis by elemental analyzer, the results of bulk analysis of total sulfur concentration, and sulfur isotope ratios[19]. The results are shown in Figure 1. The results listed for other Ryugu particles[3,33] are for larger particles that were analyzed by X-ray fluorescence (XRF) and ICP-MS. The XRF and ICP-MS results are similar to the sulfur concentration of the CI group reported by Lodders (2021)[8]. The results from Yokoyama et al. (2023)[26] are listed with XRF analysis error. One standard deviation (1SD) values are provided for the variation of sulfur concentrations of multiple particles, the results of Nakamura et al. (2022)[33], and the estimation error of Lodders (2021)[8]. *Yokoyama et al. (2023) conducted XRF analysis using two different XRF instruments.

	**Weight (µg)**	**1s**	**Total S (wt%)**	**1s**	**δ^{34}S (‰ vs. VCDT)**	**1s**
A0106 Initial Bulk run 1	14.5		2.57		-1.06	
A0106 Initial Bulk run 2	28.2		3.90		-2.44	
A0106 Initial Bulk run 3	31.2		3.32		-5.62	
**Average (*n* = 3)**	**24.6**	**±8.9**	**3.27**	**±0.67**	**-3.04**	**±2.34**
C0107 Initial Bulk run4	29.8		5.77		-2.5	
C0107 Initial Bulk run5	18.5		5.25		1.7	
C0107 Initial Bulk run6	16.2		5.07		-1.5	
C0107 Initial Bulk run7	17.8		4.76		-2.0	
C0107 Initial Bulk run8	20.6		6.49		-1.2	
**Average (*n* = 5)**	**20.6**	**±5.4**	**5.47**	**±0.68**	**-1.10**	**±1.62**
Yokoyama et al., 2023						
C0108	24 mg		4.99 ± 0.03 / 6.26 ± 0.05*			
Nakamura et al., 2022						
A0022, A0033, A0048, A0078	1.8-6.3 mg		5.50	±0.14		
C0008, C0019, C0027, C0053, C0079, C0081, C0082	2.2-10.0 mg		6.03	±0.54		
CI Orgueil			4.95	±0.66		
Lodders, 2021						
CI group			5.36	±0.22		

Supplementary Table 2 Blank subtracted abundances of anions and cations in the sequential extracts of Ryugu (A0106 and C0107), Orgueil, and baked serpentine. Blank cells are due to the detection limits and background from the solvent reagents. Errors are two standard deviations (2SD) of triplicate analysis.

	NH_4^+ µmol/g	2SD	Cl^- µmol/g	2SD	NO_3^- µmol/g	2SD	PO_4^{2-} µmol/g	2SD	SO_4^{2-} µmol/g	2SD	$S_2O_3^{2-}$ µmol/g	2SD
**#7-1 Hot H_2O extract**												
Ryugu A0106	0.18	0.12	41.91	1.57	11.35	2.41	D.L.		70.39	3.38	26.35	1.29
Ryugu C0107	D.L.		48.98	1.38	7.08	1.47	D.L.		98.49	1.71	37.87	1.49
CI Orgueil	33.54	6.16	15.19	0.16	D.L.		D.L.		659.10	3.82	2.91	0.12
Baked serpentine	0.17	0.10	4.26	1.20	22.30	3.76	D.L.		1.97	0.46	D.L.	
**#8 MeOH+DCM extract**												
Ryugu A0106	D.L.	D.L.	D.L.		D.L.		D.L.		0.45	0.06	D.L.	
Ryugu C0107	0.03	0.02	D.L.		D.L.		D.L.		0.22	0.06	D.L.	
CI Orgueil	D.L.	D.L.	D.L.		D.L.		D.L.		D.L.		D.L.	
Baked serpentine	0.03	0.01	D.L.		D.L.		1.56	0.37	0.47	0.01	D.L.	
**#9 HCOOH extract**												
Ryugu A0106	D.L.		D.L.		D.L.		8.66	2.70	24.47	1.55	D.L.	
Ryugu C0107	D.L.		D.L.		D.L.		8.76	1.58	19.71	1.73	D.L.	
CI Orgueil	D.L.		D.L.		D.L.		D.L.		16.68	0.28	D.L.	
Baked serpentine	D.L.		D.L.		D.L.		D.L.		1.15	0.37	D.L.	
**#10 HCl extract**												
Ryugu A0106	D.L.		ND		D.L.		2.79	0.73	3.79	0.96	D.L.	
Ryugu C0107	D.L.		ND		D.L.		3.97	1.05	5.13	0.56	D.L.	
CI Orgueil	D.L.		ND		D.L.		D.L.		25.92	0.43	D.L.	
Baked serpentine	D.L.		ND		3.29		2.64	0.97	D.L.		D.L.	
**#2 Hex extract (DW of LL separation)**												
Ryugu A0106	0.001	0.0002	D.L.		D.L.		D.L.		D.L.		D.L.	
Ryugu C0107	0.001	0.0002	D.L.		D.L.		D.L.		D.L.		D.L.	
CI Orgueil	D.L.		D.L.		D.L.		D.L.		D.L.		D.L.	
Baked serpentine	D.L.		D.L.		D.L.		D.L.		D.L.		D.L.	
**#3 DCM extract (DW of LL separation)**												
Ryugu A0106	D.L.		D.L.		D.L.		D.L.		D.L.		D.L.	
Ryugu C0107	0.002	0.0005	D.L.		D.L.		D.L.		D.L.		D.L.	
CI Orgueil	0.006	0.0026	D.L.		7.51		D.L.		D.L.		D.L.	
Baked serpentine	0.003	0.0005	D.L.		–		D.L.		D.L.		D.L.	
**#4 MeOH extract (DW of LL separation)**												
Ryugu A0106	0.001	0.0001	D.L.		1.73		D.L.		0.31		D.L.	
Ryugu C0107	0.004	0.0006	D.L.		2.57		D.L.		0.44		D.L.	
CI Orgueil	0.003	0.0005	3.76		5.65		D.L.		136.66		D.L.	
Baked serpentine	0.001	0.0001	D.L.		3.94		D.L.		0.18		D.L.	
**#5 H_2O extract**												
CI Orgueil	23.31	3.14	D.L.		D.L.		D.L.		427.08		D.L.	

Supplementary Table 2 (cont.)

	Na µmol/g	2SD	Mg µmol/g	2SD	Al µmol/g	2SD	K µmol/g	2SD	Ca µmol/g	2SD	Mn µmol/g	2SD	Fe µmol/g	2SD	Ni µmol/g	2SD
**#7-1 Hot H_2O extract**																
Ryugu A0106	405.04	22.24	19.45	0.78	D.L.		20.95	1.45	10.68	1.38	0.04	0.01	0.16	0.12	D.L.	
Ryugu C0107	563.89	18.27	34.33	5.78	D.L.		27.90	0.71	8.60	1.50	0.08	0.04	0.37	0.12	0.31	0.03
CI Orgueil	261.53	6.92	527.08	20.98	D.L.		13.63	0.45	192.39	17.48	5.08	0.21	0.01	0.01	1.79	0.06
Baked serpentine	12.10	1.04	293.60	21.98	9.93	3.04	2.31	0.18	–		0.25	0.02	12.72	1.53	0.14	0.02
**#8 MeOH+DCM extract**																
Ryugu A0106	D.L.		D.L.		D.L.		4.83	0.39	D.L.		D.L.		0.13	0.15	D.L.	
Ryugu C0107	D.L.		D.L.		D.L.		4.10	0.25	D.L.		D.L.		D.L.		D.L.	
CI Orgueil	D.L.		D.L.		D.L.		D.L.		D.L.		D.L.		D.L.		D.L.	
Baked serpentine	D.L.		D.L.		D.L.		3.20	0.16	D.L.		D.L.		D.L.		D.L.	
**#9 HCOOH extract**																
Ryugu A0106	154.57	4.78	886.38	40.99	13.09	0.60	4.03	0.40	91.33	32.29	14.93	0.78	380.50	11.31	36.09	0.70
Ryugu C0107	121.10	2.55	763.71	30.96	3.59	0.41	2.81	0.29	112.88	27.32	15.97	0.85	307.55	11.84	27.49	0.78
CI Orgueil	25.73	0.44	127.40	5.12	2.14	1.00	0.80	0.06	30.76	19.80	11.44	1.62	473.80	35.76	44.57	7.07
Baked serpentine	90.20	2.20	43.53	2.19	12.35	1.06	0.78	0.06	–		0.18	0.02	6.07	0.47	0.06	0.00
**#10 HCl extract**																
Ryugu A0106	43.08	3.09	1944.85	52.90	184.94	10.46	4.28	0.26	144.02	28.24	19.25	0.63	1263.93	36.94	34.29	0.71
Ryugu C0107	38.89	3.63	1925.78	76.84	195.65	12.93	3.65	0.63	95.12	17.23	18.73	1.31	1314.65	49.86	37.42	1.09
CI Orgueil	33.93	0.57	2242.09	69.90	196.81	43.24	3.74	0.25	60.30	37.03	17.46	1.05	1565.42	49.56	136.93	7.58
Baked serpentine	62.29	2.67	273.12	24.36	30.40	3.58	3.86	0.29	0.70	0.00	0.34	0.06	276.53	30.10	0.74	0.05
**#2 Hex extract (DW of LL separation)**																
Ryugu A0106	2.37	0.17	0.18	0.04	D.L.		1.79	0.29	D.L.		D.L.		D.L.		D.L.	
Ryugu C0107	2.74	0.23	0.20	0.04	0.18	0.17	2.04	0.37	D.L.		D.L.		D.L.		D.L.	
CI Orgueil	D.L.		0.27	0.01	D.L.		0.74	0.04	D.L.		0.01	0.00	D.L.		D.L.	
Baked serpentine	0.98	0.04	0.65	0.08	D.L.		2.08	0.23	4.02	2.81	–		D.L.		D.L.	
**#3 DCM extract (DW of LL separation)**																
Ryugu A0106	40.91	8.95	0.81	0.07	0.11	0.06	4.11	0.71	D.L.		D.L.		D.L.		D.L.	
Ryugu C0107	25.58	6.66	1.27	0.18	0.14	0.09	5.14	0.75	D.L.		D.L.		D.L.		D.L.	
CI Orgueil	7.31	0.46	0.25	0.01	0.10	0.01	0.18	0.02	D.L.		D.L.		D.L.		D.L.	
Baked serpentine	21.31	2.97	0.73	0.09	0.06	0.04	3.32	0.28	D.L.		–		D.L.		D.L.	
**#4 MeOH extract (DW of LL separation)**																
Ryugu A0106	21.33	1.81	1.63	0.24	0.60	0.20	12.97	3.09	D.L.		D.L.	D.L.	0.12	0.02	D.L.	
Ryugu C0107	43.81	4.27	25.01	4.45	2.12	1.01	55.84	13.74	D.L.		0.01	0.00	0.61	0.11	D.L.	
CI Orgueil	28.67	1.59	121.93	7.95	0.18	0.09	D.L.		D.L.		0.51	0.04	D.L.		1.00	0.05
Baked serpentine	10.44	0.62	1.72	0.20	1.05	0.53	7.55	1.63	16.17	3.23	D.L.		0.12	0.02	D.L.	
**#5 H_2O extract**																
CI Orgueil	225.37	4.30	250.36	5.19	D.L.	D.L.	14.44	0.44	111.89	18.05	4.24	0.13	D.L.		7.03	0.09

Supplementary Table 3 Concentrations of sulfur-containing ions (Supplementary Table 1) in weight percent in each elution fraction of A0106 and C0107 relative to total sulfur concentration (Supplementary Table 1), and their sum. The presence of sulfur in sulfate and other intermediate-oxidation-state forms is supported by X-ray absorption near-edge structure analysis of the bulk sample[34].

	SO_4^{2-}	2SD	$S_2O_3^{2-}$	2SD
	(% relative to Total S)			
**#7-1 Hot H_2O extract**				
Ryugu A0106	6.91%	0.33%	2.59%	0.13%
Ryugu C0107	5.78%	0.10%	2.22%	0.09%
**#8 MeOH+DCM extract**				
Ryugu A0106	0.04%	0.01%	DL	
Ryugu C0107	0.01%	0.00%	DL	
**#9 HCOOH extract**				
Ryugu A0106	2.40%	0.15%	DL	
Ryugu C0107	1.16%	0.10%	DL	
**#10 HCl extract**				
Ryugu A0106	0.37%	0.09%	DL	
Ryugu C0107	0.30%	0.03%	DL	
**SUM**				
Ryugu A0106	9.73%		2.59%	
Ryugu C0107	7.25%		2.22%	

Supplementary Table 4 The sum of major anions and cations listed in Supplementary Table 2 (in meq per gram of solid sample). Note that dissolved inorganic carbon and silica were not measured because of the limited amounts of available sample. "DW of LL" denotes the ions dissolved in the water (DW) phase of liquid-liquid (LL) separation.

	Σ^- Cl^-, NO_3^-, PO_4^{2-}, SO_4^{2-}, $S_2O_3^{2-}$	Σ^+ NH_4^+, Na^+, Mg^{2+}, K^+, Ca^{2+}	$\Delta\Sigma$ Cation - Anion	Total soluble-component content
	meq/g	meq/g	meq/g	mg/g
**#7-1 Hot H_2O extract**				
Ryugu A0106	0.247	0.486	0.240	23.0
Ryugu C0107	0.329	0.678	0.349	31.2
CI Orgueil	1.336	1.748	0.412	92.2
Baked serpentine	0.031	0.602	0.571	10.2
**#8 MeOH+DCM extract**				
Ryugu A0106	0.001	0.005	0.004	0.2
Ryugu C0107	0.000	0.004	0.004	0.2
CI Orgueil	0.000	0.000	0.000	0.0
Baked serpentine	0.006	0.003	-0.002	0.3
**#9 HCOOH extract**				
Ryugu A0106	0.075	2.114	2.039	56.6
Ryugu C0107	0.066	1.877	1.811	48.5
CI Orgueil	0.033	0.343	0.310	36.3
Baked serpentine	0.002	0.173	0.170	4.0
**#10 HCl extract**				
Ryugu A0106	0.016	4.225	4.209	133.5
Ryugu C0107	0.022	4.084	4.062	134.4
CI Orgueil	0.052	4.642	4.591	162.1
Baked serpentine	0.011	0.614	0.603	25.0
**Sum of #7-1, #8, #9, and #10**				
Ryugu A0106				213.3
Ryugu C0107				214.3
CI Orgueil				290.6
Baked serpentine				39.5
**#2 Hex extract (DW of LL separation)**				
Ryugu A0106	0.000	0.005	0.005	0.3
Ryugu C0107	0.000	0.005	0.005	0.3
CI Orgueil	0.000	0.001	0.001	0.2
Baked serpentine	0.000	0.012	0.012	0.4
**#3 DCM extract (DW of LL separation)**				
Ryugu A0106	0.000	0.047	0.047	1.2
Ryugu C0107	0.000	0.033	0.033	1.0
CI Orgueil	0.008	0.008	0.000	0.9
Baked serpentine	0.000	0.026	0.026	0.8
**#4 MeOH extract (DW of LL separation)**				
Ryugu A0106	0.002	0.038	0.035	1.2
Ryugu C0107	0.003	0.150	0.146	4.1
CI Orgueil	0.283	0.273	-0.010	17.3
Baked serpentine	0.004	0.054	0.049	1.5
**#5 H_2O extract**				
CI Orgueil	0.854	0.988	0.133	60.6

Supplementary Table 5 The percentages of clay mineral and carbonate standards dissolved in the sequential extraction acids of Naraoka et al. (2023)[2]. The percentage of dissolution was calculated by measuring Al or Mg for clay minerals and Ca for carbonates. The ratio of solid to liquid phase of clay minerals is the same as the solid/liquid ratio of Naraoka et al. (2023)[2], with 600 μL of reagent added for 15 mg of solids. As the abundance of carbonates in Ryugu is a few percent, the sample weight of dolomite experiment was reduced to 1.5 mg of sample.

Reagent type	20% HCl		>99% HCOOH		6% CH_3COOH		0.25M EDTA-Na
Fraction number of Naraoka+2023	(#10)		(#9)				
Percentage dissolved	%	2SD	%	2SD	%	2SD	%
**Clay**							
Condition 1: 15 mg clay + 600 μL reagents							
JCSS-3501 Saponite	103.3%	2.3	10.6%	0.3	9.7%	0.1	<0.1
JCSS-3101b Montmorillonite	3.1%	0.1	0.5%	<0.1	1.6%	<0.1	<0.1
JCSS-2101 Pyrophyllite	0.6%	2.4	0.3%	<0.1	0.6%	<0.1	<0.1
JCSS-1301 Dickite	0.9%	<0.1	0.2%	<0.1	0.5%	<0.1	<0.1
JCSS-1101c Kaolinite	10.1%	0.2	1.6%	<0.1	2.6%	0.1	<0.1
Condition 2: 1 mg clay + 600 μL reagents							
JCSS-3501 Saponite	97.7%	2.5	-		-		-
JCSS-3101b Montmorillonite	6.6%	0.8	-		-		-
Condition 3: 75 mg clay + 600 μL reagents							
JCSS-3501 Saponite	80.6%	2.3	-		-		-
JCSS-3101b Montmorillonite	5.3%	0.3	-		-		-
**Carbonate**							
AIST JDo-1 Dolomite	-		103.3%	2.6	-		-

Supplementary Table 6 Blank subtracted abundances of cations in the sequential extracts of reference carbonaceous meteorites (CI1 Orgueil, $C2_{ung}$ Tarda, and CM2 Aguas Zarcas and Jbilet Winselwan).

	Na	2SD	Mg	2SD	Al	2SD	K	2SD	Ca	2SD	Mn	2SD	Fe	2SD
	μmol/g		μmol/g		μmol/g		μmol/g		μmol/g		μmol/g		μmol/g	
**#9 HCOOH extract**														
C2 Tarda	118.41	1.99	1413.63	44.07	-		11.17	0.75	21.90	26.89	9.05	0.55	129.15	4.09
CM2 Aguas	41.80	2.11	1787.96	50.35	-		5.48	0.45	61.81	52.59	10.68	0.18	210.02	10.16
CM2 Jbilet	124.77	2.67	1107.84	41.01	-		45.10	2.38	551.71	98.95	12.42	0.23	182.57	6.24
**#10 HCl extract**														
C2 Tarda	104.80	2.15	10614.13	263.58	979.56	31.62	10.66	1.08	647.24	250.75	71.52	1.66	7121.17	66.19
CM2 Aguas	87.11	2.90	7252.74	331.79	924.69	40.48	9.22	0.78	194.11	72.09	52.51	2.78	8996.43	404.70
CM2 Jbilet	201.21	3.61	8767.06	238.30	1147.19	15.70	17.40	0.98	104.39	51.87	62.09	2.94	11282.29	301.55

Supplementary Table 7. Ammonia and amine abundances in hot water extracts of Ryugu A0106, C0107, and Orgueil. For reference, the amount of NH_3 released by hydrothermal treatment (300 °C and 100 MPa) of the insoluble organic material of Orgueil is ~19 μmol/g. (ref. 25). A significant amount of NH_3 is also present only in salt-bound form in Orgueil, not in the organic, carbonate, or phyllosilicate fractions (Supplementary Table 2). Errors shown are 1SD.

**Species**	**Ryugu A0106 (nmol/g)**	**Ryugu C0107 (nmol/g)**	**Orgueil (nmol/g)**	**Orgueil/A106 ratio**	**Orgueil/C107 ratio**	***p*K (at 25°C)**	**Vapor Pressure (kPa @ 20°C)**
**Ammonia**	180±60	<20	33540±3080	190±24	>1677	9.2	857
**Methylamine**	23.79±0.64 [a,b]	34.14±1.74 [b]	331.5±0.2 [c]	13.93±0.37	9.71±0.49	10.7	186
**Ethylamine**	11.37±0.34[a,b]	10.70±1.98 [b]	27.3±2.4 [c]	2.40±0.22	2.55±0.52	10.8	116
**Isopropylamine**	0.59±0.03[a,b]	0.62±0.06 [b]	4.8±0.1[c]	8.13±0.12	7.74±0.77	10.7	33.0
***n*-Propylamine**	0.05±0.01[a,b]	0.08±0.02 [b]	5.1±0.04 [c]	100±20	64±16	10.6	63.4

[a] Naraoka et al. (2023)[2]

[b] Parker et al. (2023)[17]

[c] Aponte et al. (2016)[35]

Supplementary Table 8 Magnesium isotope ratios of the leacheates, normalized as $\delta^{26}Mg$ and $\delta^{25}Mg$ (‰, vs. the DSM-3 standard[13]) within two standard deviations (2SD) of triplicate analysis. The pure Mg standard Cambridge-1[13], which does not require column separation, was also measured.

Sample	$\delta^{26}Mg$	2SD	$\delta^{25}Mg$	2SD
Orgueil, #7-1 hot H_2O	-1.49	0.06	-0.77	0.01
A0106, #9 HCOOH	-0.56	0.07	-0.29	0.05
C0107, #9 HCOOH	-0.65	0.07	-0.36	0.09
Orgueil, #9 HCOOH	-1.32	0.09	-0.65	0.06
Tarda, #9 HCOOH	-0.77	0.07	-0.38	0.03
Aguas Zarcas, #9 HCOOH	-0.66	0.07	-0.34	0.03
Jbilet Winselwan, #9 HCOOH	-0.56	0.07	-0.28	0.05
A0106, #10 HCl	-0.26	0.09	-0.19	0.05
C0107, #10 HCl	-0.24	0.07	-0.14	0.02
Orgueil, #10 HCl	-0.07	0.07	-0.06	0.05
Tarda, #10 HCl	-0.19	0.06	-0.08	0.04
Aguas Aguas, #10 HCl	-0.21	0.06	-0.08	0.04
Jbilet Winselwan, #10 HCl	-0.22	0.09	-0.10	0.05
Orgueil-#5	-1.42	0.08	-0.75	0.03
Orgueil-#7-2	-0.16	0.08	-0.09	0.08
Serpentine-#9	-0.42	0.07	-0.24	0.03
Serpentine-#10	-0.30	0.19	-0.19	0.02
Cambridge 1 (n = 9)	-2.61	0.05	-1.35	0.04

References

1. Miyawaki, R. et al. Some Reference Data for the JCSS Clay Specimens. *J. Clay Sci. Soc. Jpn.* **48**, 158–198 (2010). (in Japanese with English abstract)
2. Naraoka, H. et al. Soluble organic molecules in samples of the carbonaceous asteroid (162173) Ryugu. *Science* **379**, eabn9033 (2023).
3. Nakamura, T. et al. Formation and evolution of carbonaceous asteroid Ryugu: Direct evidence from returned samples. *Science* **379**, eabn8671 (2023).
4. Fedo, C.M., Wayne Nesbitt, H., & Young, G.M. Unraveling the effects of potassium metasomatism in sedimentary rocks and paleosols, with implications for paleoweathering conditions and provenance. *Geology* **23**, 921–924 (1995).
5. Clarke Jr, R.S. et al. Allende, Mexico, meteorite shower. *Smithsonian Contributions to the Earth Sciences* **5** (1971).
6. Fuchs, L.H., Olsen, E. & Jensen, K.J. Mineralogy, mineral-chemistry, and composition of the Murchison (C2) meteorite. *Smithsonian Contributions to the Earth Sciences* **10** (1973).
7. Wiik, H.B. The chemical composition of some stony meteorites. *Geochim. Cosmochim. Acta* **9**, 279–289 (1956).
8. Lodders, K. Relative atomic solar system abundances, mass fractions, and atomic masses of the elements and their isotopes, composition of the solar photosphere, and compositions of the major chondritic meteorite groups. *Space Sci. Rev.* **217**, Article number: 44 (2021).
9. Nesbitt, H. & Young, G. Early Proterozoic climates and plate motions inferred from major element chemistry of lutites. *Nature* **299**, 715–717 (1982).
10. Browning, L.B., McSween Jr, H.Y. & Zolensky, M.E. Correlated alteration effects in CM carbonaceous chondrites. *Geochim. Cosmochim. Acta* **60**, 2621–2633 (1996).
11. Li, W., Beard, B.L. & Johnson, C.M. Exchange and fractionation of Mg isotopes between epsomite and saturated $MgSO_4$ solution. *Geochim. Cosmochim. Acta* **75**, 1814–1828 (2011).
12. Pearce, C.R., Saldi, G.D., Schott, J. & Oelkers, E.H. Isotopic fractionation during congruent dissolution, precipitation and at equilibrium: Evidence from Mg isotopes. *Geochim. Cosmochim. Acta* **92**, 170–183 (2012).
13. Teng, F.Z. Magnesium isotope geochemistry. *Rev. Mineral. Geochem.* **82**, 219–287 (2017).
14. Teng, F.Z. et al. Magnesium isotopic composition of the Earth and chondrites. *Geochim. Cosmochim. Acta* **74**, 4150–4166 (2010).
15. Tachibana, S. et al. Pebbles and sand on asteroid (162173) Ryugu: In situ observation and particles returned to Earth. *Science* **375**, 1011–1016 (2022).

16. Kminek, G. & Bada, J.L. The effect of ionizing radiation on the preservation of amino acids on Mars. *Earth Planet. Sci. Lett.* **245**, 1–5 (2006).
17. Parker, E.T. et al. Extraterrestrial Amino Acids and Amines Identified in Asteroid Ryugu Samples Returned by the Hayabusa2 Mission. *Geochim. Cosmochim. Acta* **347**, 42–57 (2023).
18. Oba, Y. et al. Identifying the wide diversity of extraterrestrial purine and pyrimidine nucleobases in carbonaceous meteorites. *Nat. Commun.* **13**, Article number: 2008 (2022).
19. Oba, Y. et al. Uracil in the carbonaceous asteroid (162173) Ryugu. *Nat. Commun.* **14**, Article number: 1292 (2023).
20. Okazaki, R. et al. First asteroid gas sample delivered by the Hayabusa2 mission: A treasure box from Ryugu. *Sci. Adv.* **8**, eabo7239 (2022).
21. Okazaki, R. et al. Noble gases and nitrogen in samples of asteroid Ryugu record its volatile sources and recent surface evolution. *Science* **379**, eabo0431 (2023).
22. Ehlmann, B.L. et al. Ambient and cold-temperature infrared spectra and XRD patterns of ammoniated phyllosilicates and carbonaceous chondrite meteorites relevant to Ceres and other solar system bodies. *Meteorit. Planet. Sci.* **53**, 1884–1901 (2018).
23. Aponte, J.C., Dworkin, J.P. & Elsila, J.E. Indigenous aliphatic amines in the aqueously altered Orgueil meteorite. *Meteorit. Planet. Sci.* **50**, 1733–1749 (2015).
24. Altwegg, K. et al. Evidence of ammonium salts in comet 67P as explanation for the nitrogen depletion in cometary comae. *Nat. Astron.* **4**, 533–540 (2020).
25. Pizzarello, S. & Williams, L. Ammonia in the early solar system: An account from carbonaceous meteorites. *Astrophys. J.* **749**, 161 (2012).
26. Yokoyama, T. et al. Samples returned from the asteroid Ryugu are similar to Ivuna-type carbonaceous meteorites. *Science* **379**, eabn7850 (2023).
27. Khoo, K., Culberson, C.H. & Bates, R.G. Thermodynamics of the dissociation of ammonium ion in seawater from 5 to 40 °C. *J. Solution Chem.* **6**, 281–290 (1977).
28. Yada, T. et al. Preliminary analysis of the Hayabusa2 samples returned from C-type asteroid Ryugu. *Nat. Astron.* **6**, 214–220 (2022).
29. Sakamoto, K. et al. Environmental assessment in the prelaunch phase of Hayabusa2 for safety declaration of returned samples from the asteroid (162173) Ryugu: Background monitoring and risk management during development of the sampler system. *Earth Planets Space* **74**, Article number: 90 (2022).
30. Yoshitake, M. et al. *Cleanliness level of the Extraterrestrial Sample Curation Center of JAXA. JAXA Technical Report*. JAXA-RR-20-004E (JAXA, 2021). doi:10.20637/00047360.

31. Yada, T. et al. A curation for uncontaminated Hayabusa2-returned samples in Extraterrestrial Curation Center of JAXA: From the beginning to nowadays. *Earth Planets Space* (2023, in revision).
32. Schmitt-Kopplin, P. et al. High molecular diversity of extraterrestrial organic matter in Murchison meteorite revealed 40 years after its fall. *Proc. Natl. Acad. Sci. USA* **107**, 2763–2768 (2010).
33. Nakamura, E. et al. On the origin and evolution of the asteroid Ryugu: A comprehensive geochemical perspective. *Proc. Jpn. Acad. B* **98**, 227–282 (2022).
34. Suga, H. et al. Sulfur-XANES of intact Ryugu grains and the isolated IOM. *Hayabusa 2022 Symposium*, # Abstract S32-02 (2022).
35. Aponte, J.C., McLain, H.L., Dworkin, J.P. & Elsila, J.E. Aliphatic amines in Antarctic CR2, CM2, and CM1/2 carbonaceous chondrites. *Geochim. Cosmochim. Acta* **189**, 296–311 (2016).

List of Hayabusa2-initial-analysis SOM team members

Hiroshi Naraoka[2*], Yoshinori Takano[1], Jason P. Dworkin[7], Kenji Hamase[20], Aogu Furusho[20], Minako Hashiguchi[10], Kazuhiko Fukushima[21], Dan Aoki[21], José C. Aponte[7], Eric T. Parker[7], Daniel P. Glavin[7], Hannah L. McLain[7,22,23], Jamie E. Elsila[7], Heather V. Graham[7], John M. Eiler[24], Philippe Schmitt-Kopplin[4,5], Norbert Hertkorn[4], Alexander Ruf[25,26,27], Francois-Regis Orthous-Daunay[28], Cédric Wolters[28], Junko Isa[29,30], Véronique Vuitton[28], Roland Thissen[31], Nanako O. Ogawa[1], Saburo Sakai[1], Toshihiro Yoshimura[1], Toshiki Koga[1], Haruna Sugahara[17], Naohiko Ohkouchi[1], Hajime Mita[32], Yoshihiro Furukawa[33], Yasuhiro Oba[6], Yoshito Chikaraishi[6], Takaaki Yoshikawa[8], Satoru Tanaka[9], Mayu Morita[34], Morihiko Onose[34], Daisuke Araoka[3], Fumie Kabashima[35], Kosuke Fujishima[29], Hajime Sato[36], Kazunori Sasaki[36,37], Kuniyuki Kano[38], Shin-ichiro M. Nomura[39], Junken Aoki[38], Tomoya Yamazaki[6], Yuki Kimura[6].

* HY2 SOM consortium representative, Email: naraoka@geo.kyushu-u.ac.jp

[20] Graduate School of Pharmaceutical Sciences, Kyushu University, Fukuoka 812-8582, Japan.

[21] Graduate School of Bioagricultural Sciences, Nagoya University, Nagoya 464-8601, Japan.

[22] Center for Research and Exploration in Space Science and Technology, NASA Goddard Space Flight Center, Greenbelt, MD 20771, USA.

[23] Department of Physics, The Catholic University of America, Washington, DC 20064, USA.

[24] Division of Geological and Planetary Sciences, California Institute of Technology, Pasadena, CA 91125, USA.
[25] Université Aix-Marseille, CNRS, Laboratoire de Physique des Interactions Ioniques et Moléculaires, Marseille 13397, France.
[26] Department of Chemistry and Pharmacy, Ludwig-Maximilians-University, Munich 81377, Germany.
[27] Excellence Cluster ORIGINS, Garching 85748, Germany.
[28] Université Grenoble Alpes, Centre National de la Recherche Scientifique (CNRS), Centre National d'Etudes Spatiales, L'Institut de Planétologie et d'Astrophysique de Grenoble, Grenoble 38000, France.
[29] Earth-Life Science Institute (ELSI), Tokyo Institute of Technology, Tokyo 152-8550, Japan.
[30] Planetary Exploration Research Center, Chiba Institute of Technology, Narashino 275-0016, Japan.
[31] Université Paris-Saclay, CNRS, Institut de Chimie Physique, Orsay 91405, France.
[32] Department of Life, Environment and Material Science, Fukuoka Institute of Technology, Fukuoka 811-0295, Japan.
[33] Department of Earth Science, Tohoku University, Sendai 980-8578, Japan.
[34] HORIBA Techno Service Co., Ltd., Kyoto, 601-8305, Japan.
[35] LECO Japan Corp., Tokyo, 105-0014, Japan.
[36] Institute for Advanced Biosciences (IAB), Keio University, Kakuganji, Tsuruoka, Yamagata 997-0052, Japan.
[37] Human Metabolome Technologies (HMT) Inc., Kakuganji, Tsuruoka, Yamagata 997-0052, Japan.
[38] Department of Health Chemistry, Graduate School of Pharmaceutical Sciences, The University of Tokyo, Hongo, Tokyo 113-0033, Japan.
[39] Department of Robotics, Graduate school of Engineering, Tohoku University, Sendai, Miyagi, 980-8579, Japan.
